# A faked prolongation of an endurance target time does not affect muscle fatigue but increases perceived exertion

**DOI:** 10.3389/fpsyg.2025.1466750

**Published:** 2025-08-27

**Authors:** Max Herzberg, Jenny Rosendahl, Lena Mader, Christoph Anders

**Affiliations:** ^1^Division of Motor Research, Pathophysiology and Biomechanics, Experimental Trauma Surgery, Clinic for Trauma, Hand and Reconstructive Surgery, Jena University Hospital, Friedrich Schiller University Jena, Jena, Germany; ^2^Institute for Psychosocial Medicine, Psychotherapy and Psychooncology, Jena University Hospital, Friedrich Schiller University Jena, Jena, Germany

**Keywords:** back muscles, static endurance, EMG, rating of perceived exertion, mental fatigue

## Abstract

**Objective:**

While psychological factors are known to influence physical performance, responses to unexpectedly extended endurance tasks remain unclear.

**Methods:**

In a crossover-randomized study, 37 participants performed an isometric endurance task twice, with a 14-day interval, compensating for 50% of upper body weight for 10 min. Muscular fatigue was measured via EMG of the back muscles, and perceived exertion (RPE; Borg scale 6–20) was recorded every minute (Real). In the experimental condition (Fake), RPE was recorded every 50 s without participants’ awareness. After the tenth query, participants were told a technical error occurred and were asked to continue for two additional minutes with two extra RPE queries. Participants were grouped by RPE and Fatigue Index (FI) into “good-end” and “bad-end” groups. FI and RPE were compared between Real and Fake conditions.

**Results:**

RPE-based grouping showed no significant FI differences. FI-based grouping revealed significantly higher RPE in the “good-end” group during the Fake condition (+0.9 at 540/550 s; +1.0 at 600 s). No significant differences were found in the “bad-end” group.

**Conclusion:**

Extending a task beyond its expected endpoint increases perceived exertion, which may lead to task termination despite unchanged muscular fatigue.

## 1 Introduction

The relationship between psychological and motivational factors and physical performance, particularly during endurance exercises, has been extensively studied. For many years, the theory that the termination of endurance activities is primarily due to muscular and cardiorespiratory fatigue has been questioned. Rather, it appears that task termination is based on a decision-making process to discontinue the endurance activity (Blanchfield et al., 2014). This psychobiological model of endurance performance is grounded in the theory of motivational intensity (Brehm and Self, 1989). According to this theory, an endurance task is terminated when the required effort exceeds either the maximum effort an individual is willing to exert, or when the perceived effort is judged to have reached its limit, making continuation seem impossible (Marcora et al., 2008; Marcora et al., 2009; Marcora and Staiano, 2010; Staiano et al., 2018). Studies have shown that, especially in highly motivated individuals, the rating of perceived exertion (RPE) is the key factor for task termination (Staiano et al., 2018; Marcora, 2010).

Various theories have been proposed to explain the origin of perceived exertion. The most widely supported is the corollary discharge model (Pageaux, 2016). This model suggests that perceived exertion arises from neural processes involving corollary discharges from the cortex, which are associated with central motor control. Corollary discharges are internal signals that originate from efferent motor commands and modulate the activity of premotor and motor areas during voluntary muscle contractions, thereby influencing the perception of exertion (Pageaux, 2016). Moreover, animal studies have demonstrated that prolonged neural activity - whether due to physical or mental fatigue - leads to an increase in extracellular adenosine concentrations. This mechanism may further contribute to the rise in perceived exertion (Lovatt et al., 2012; Pageaux, 2014; Pageaux et al., 2015). Supporting this, the administration of caffeine, an adenosine antagonist, has been shown to enhance both physical and psychological performance in fatigued individuals (McLellan et al., 2016).

Furthermore, it has been demonstrated that positive self-talk during and between exercise bouts on a cycling ergometer improves performance and prolongs endurance (Blanchfield et al., 2014). Verbal encouragement has also shown beneficial effects, increasing maximum voluntary contraction by approximately 5% compared to control conditions without encouragement (McNair et al., 1996), and extending endurance time to exhaustion by 8–18%, depending on the source (Midgley et al., 2018).

In all of the aforementioned endurance testing studies, psychological interventions were introduced before the exercise task. The present study aims to investigate what occurs when a psychological stressor is applied during the exercise itself, specifically at the moment participants believe they have completed the task. To examine this, we postponed the declared end of the endurance task in one of two test conditions. This allowed us to evaluate how a virtual extension (i.e., an apparent prolongation of the task) influences both physiological fatigue, assessed via surface electromyography (SEMG), and subjective exertion measured through RPE. Since the postponed task completion was only virtual, participants in both conditions performed an isometric back muscle endurance task for a target time of 10 min at 50% of their upper body weight.

## 2 Materials and methods

### 2.1 Participants

The study employed a sex-balanced, randomized controlled crossover design. Participants were recruited via personal contacts, public notices, and electronic media. Eligibility criteria included an age range of 25 to 50 years and the absence of any spine-related complaints or history of spinal surgery. Exclusion criteria encompassed individuals outside the specified age range, those with current or chronic back pain, a Body Mass Index (BMI) below 18 or above 28, a body height less than 1.50 m or greater than 1.90 m (due to device limitations), individuals with mental or psychological impairments, and those engaged in systematic fitness training exceeding one hour on more than two days per week. A total of 37 participants (19 women) were enrolled in the study (see Table 1).

**Table 1:** Participants characteristics.

Group	Parameter	Age [years]	Height [cm]	Weight [kg]	BMI [kg/m^2^]
Men (*n* = 18)	MV	31.6	182.2	78.6	23.6
	SD	5.6	5.6	9.1	2.2
	Median	30	184	79	24
	u.q.	7	3	3	2
	l.q.	2	5	5	2
Women (*n* = 19)	MV	34.8	168.9	65.1	22.9
	SD	7.8	7.3	7.2	2.6
	Median	36	168	65	22
	u.q.	4	7	5	3
	l.q.	9	5	4	2

n, sample size; MV, mean value; SD, standard deviation, u.q., difference of medians to upper quartile, l.q., difference of medians to lower quartile.

### 2.2 Measurement procedure and device

**Figure 1:**
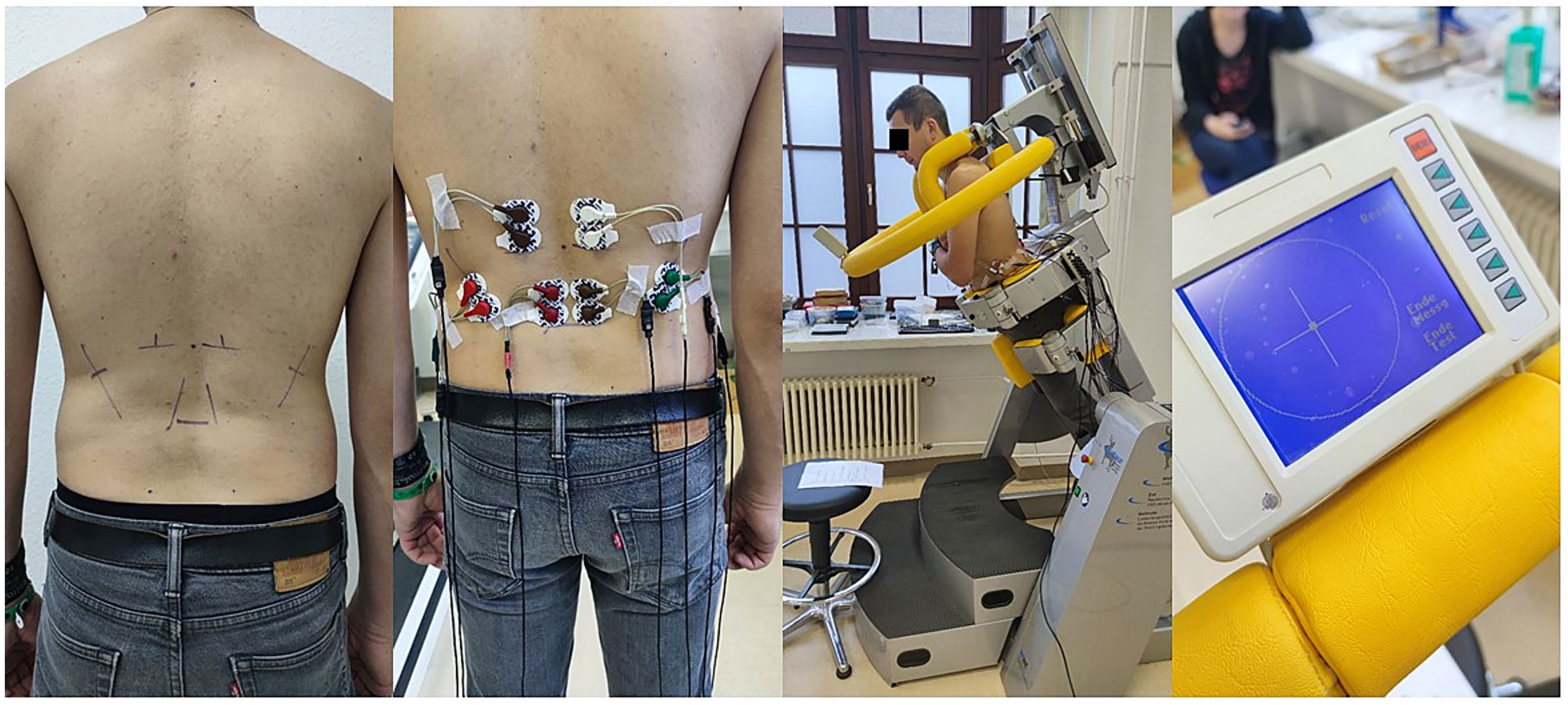
From left to right: markings for back muscles, applied electrodes and amplifiers, participant in 30° forward tilt during the endurance test, biofeedback monitor with the control point located right in the center of the crosshair.

Two measurement sessions were conducted 14 days apart at the same time of day to minimize the influence of circadian fluctuations. Based on anatomical landmarks, surface electrode positions for two back muscles—Musculus multifidus lumbalis (MF) and Musculus longissimus thoracis (LO)—were identified bilaterally (right – R, and left – L) following international guidelines (Hermens et al., 1999) (see Figure 1). Although the Musculus iliocostalis was also measured, its data were excluded from analysis, as its primary force vector cannot be clearly assigned to either anterior or posterior force directions (Anders and Steiniger, 2018).

All electrode positions were marked by the same experienced investigator (CA). If required, the marked areas were shaved to ensure secure attachment of the self-adhesive electrodes. The skin was then prepared using an abrasive paste (Nihon Kohden) (Hermens et al., 2000), and Ag/AgCl disposable electrodes (H39 SG, Covidien, Germany) were applied with an inter-electrode distance of 2.5 cm (see Figure 1), and connected to amplifiers (Biovision, Germany). Additionally, four data channels were recorded directly from the testing device (CTT Centaur, BfMC, Germany), capturing tilt and rotation angles as well as forces measured in the x-and y-directions via integrated force sensors.

To minimize the influence of electrode repositioning on measurement variability, participants were instructed not to remove the position markings, but to renew them before the second session. At the second appointment, these markings were checked, renewed, and corrected if necessary by the same investigator.

Following electrode placement, participants were positioned in the CTT Centaur testing device, which enables the application of defined forces to the upper body through adjustable rotation angles (−180° to +180°) and tilt angles (0° to 90°). Participants were secured from the hips downward. A bar equipped with force sensors (x and y axes) was placed across their shoulders, allowing limited upper-body movement. The task required participants to stabilize their upper body along the longitudinal axis and maintain an upright posture. Arms were held crossed in front of the torso.

A small biofeedback monitor, visible to the participant, displayed a crosshair and a movable point. Any force applied to the bar caused the point to shift from the center of the crosshair, indicating direction and magnitude. This allowed both the participant and examiner to continuously monitor and correct posture throughout the measurement.

### 2.3 Measurement procedure: real and fake situations

To control for potential gender-related motivational influences, all instructions were delivered by an experimenter of the opposite sex to the participant. This methodological approach was based on studies suggesting that experimenter-participant gender dynamics can affect participant responses (Chapman et al., 2018; Singer and Llewellyn, 1973). Any questions from participants were addressed accordingly.

Following a defined submaximal warm-up protocol within the testing device, participants were tilted forward at a 30° angle and instructed to align their upper body with the body’s longitudinal axis, maintaining an upright posture for 10 min. In this position, they were required to compensate exactly 50% of their own upper body weight due to gravitational load. Care was taken to prevent any involuntary muscle activation, such as speaking or laughing that could influence abdominal or back muscle activity during task execution. A hidden timer was used during the measurement period, allowing RPE (Rating of Perceived Exertion) to be recorded using the Borg scale (6 to 20) (Borg, 1982) after each elapsed minute. Participants were informed of this procedure in advance. This condition is referred to as the “Real” trial.

In one of the two measurement sessions (sequence sex-balanced and randomized: two flipped stacks of equally distributed sequences), RPE was queried every 50 s instead of every 60 s, without informing the participants. After ten queries - believed by participants to represent 10 min - they assumed the task was complete, although only 8 min and 20 s had actually passed. Participants were then informed that the recording system had likely failed to register the first two minutes or had experienced an unnoticed interruption which was not noticed by the measurement team. As a result, they were told the measurement needed to continue for an additional two minutes to achieve a full 10-min data set, which was presented as essential for ensuring data comparability. This condition is referred to as the “Fake” trial.

Participants had been previously informed and had provided written consent for participation in what was described as a reliability study, supporting the explanation for the necessity of a 10-min measurement duration. During the virtually extended period, two additional RPE values were recorded until the full 10-min time was completed. After the second session, participants were debriefed regarding the deception and were required to reconfirm their written consent for data use. The entire procedure was reviewed and approved by the Ethics Committee of the University Hospital Jena (reference: 2021-2373-BO).

### 2.4 SEMG data signal recording

The recorded SEMG signals were amplified (gain: 1000; input impedance: 1200 GΩ; noise level: < 1 μV; bandwidth: 10–700 Hz; first-order RC filter; Biovision, Germany), and subsequently analog-to-digital converted at a sampling rate of 2048 Hz (Tower of Measurement (ToM), DeMeTec, Germany). The system featured 24-bit resolution at ±5 V, corresponding to 0.6 nV/bit, and included an anti-aliasing filter set at 1024 Hz. The data were acquired using ATISArec software (GJB Datentechnik, Germany) and stored for offline analysis.

All acquired signals were continuously monitored during data collection for baseline noise, mains interference (50 Hz hum), and overall signal integrity. Electrodes or amplifiers were replaced as necessary to maintain signal quality throughout the measurement process.

### 2.5 Data processing

The recorded digital SEMG signals were subsequently reviewed using ATISApro software (GJB Datentechnik, Germany), and the 600-s period from the onset of the steady-state phase at a 30° forward tilt was identified. The data were then quantified for further analysis using MATLAB (The Mathworks, US) and stored in Excel for subsequent processing. Parameter analysis was conducted with a 0.25-s overlap for time windows of 0.5 s, continuously throughout the entire measurement duration. For each interval, the power spectrum was computed using fast Fourier transformation (FFT) (Chatfield, 1982), and the spectral fatigue parameter FI_nsm5_ (fatigue index normalizing spectral moment of order 5; FI) (Dimitrov et al., 2006) was calculated.

### 2.6 Group division into good end and bad end

In the analysis of the entire group, no significant differences were observed in either the SEMG or Borg data when comparing the Real and Fake trials. Therefore, a *post hoc* analysis was performed, creating two subgroups: good end (less fatigued) and bad end (highly fatigued) for further comparison. This classification was done twice: once based on the rating of perceived exertion (RPE), a subjectively perceived parameter, and once based on the fatigue index (FI), an objectively measurable parameter.

For the RPE-based classification, participants’ scores on the Borg scale were used. Participants who reported RPE levels of 18 or higher at the end of the Real trial were assigned to the bad end group (*n* = 13), while those reporting RPE levels of 14 or lower were assigned to the good end group (*n* = 12). Based on this classification FI changes over time were compared between Real and Fake situations, separately for every investigated muscle.

To define the groups using FI, the mean out of the values of Musculus multifidus lumbalis (MF) and Musculus longissimus thoracis (LO) for both left (L) and right (R) sides in the Real trial were used. The relative change in these values, normalized to the mean of the first 20 s, was calculated. The group was then divided into thirds based on the relative change at 600 s. The 12 participants exhibiting the largest differences were classified into the bad end group, while the 12 participants with the smallest differences were assigned to the good end group. Based on this classification RPE changes over time were compared between Real and Fake situations for those time points which were identical or deviated for maximal ten seconds (see 2.7.2).

**Figure 2:**
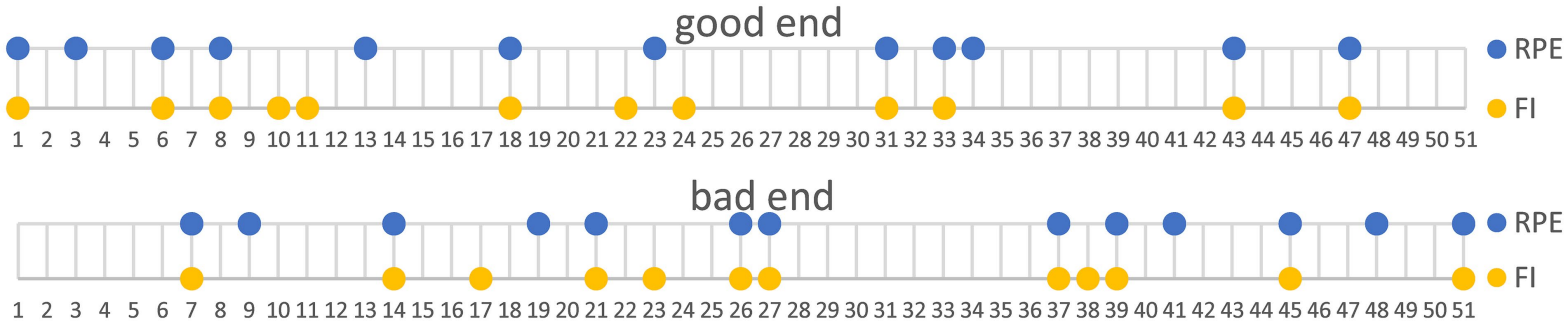
Division of participants into groups, top: good end, bottom: bad end based on rating of perceived exertion (RPE, yellow) or fatigue index (FI, blue).

In Figure 2, participant numbers are shown on the x-axis, organized according to the two groups formed. If a participant is represented by two points (e.g., participant no. 6), it indicates that they were classified into both the good end or bad end groups consistently for both RPE and FI classifications.

### 2.7 Statistical analysis

#### 2.7.1 SEMG

The analysis of the FI was performed within the good end and bad end groups, as classified according to the RPE. FI measurements were examined for each minute of the trial. For each completed minute, the average value of the measurements within a 10-s window was calculated, and a paired t-test was conducted for comparison. No correction for multiple testing was applied, as every test could have been analyzed separately. Additionally, standardized mean differences, or effect sizes (Cohen’s *d*) (Cohen, 1992), were computed to compare the Real and Fake trials. For clarity only those significant differences will be reported which are accompanied by effect sizes >0.5.

#### 2.7.2 RPE

The analysis of the RPE was conducted within the good end and bad end groups, as classified according to the FI. Due to the differing timing of the RPE queries, not all values could be directly compared between the good end and bad end groups. Therefore, only values recorded at identical times (300 and 600 s) and those within a 10-s window (50/60 s, 240/250 s, and 540/550 s) were compared. These comparisons were performed using a paired t-test for the Real vs. Fake conditions, and effect sizes (Cohen’s *d*) (Cohen, 1992) were calculated. No correction for multiple testing was applied, as every test could have been analyzed separately. For clarity only those significant differences will be reported which are accompanied by effect sizes >0.5.

## 3 Results

### 3.1 Group comparisons for FI based on extreme RPE groups

**Figure 3:**
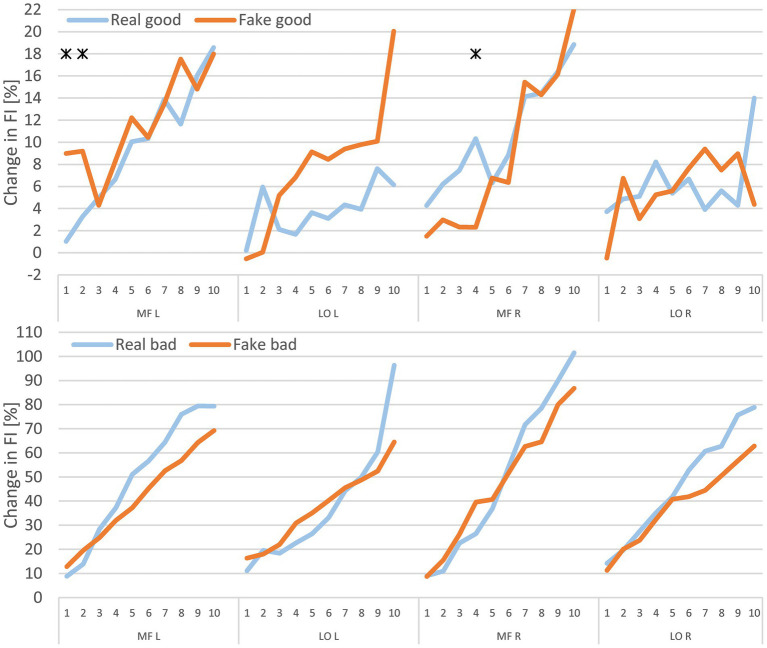
Mean percentage changes in fatigue index (FI) for each minute in real and fake, based on the extreme RPE groups. Top, good end; bottom, bad end; * indicates *p* < 0.05 and Cohen’s *d* > 0.5. MF, multifidus muscle; LO, longissimus muscle; L, left; R, right.

The classification of good end and bad end participants for the FI comparison was based on RPE. Significant differences in FI were observed only in the good end group. In contrast, no significant differences were found in the bad end group when comparing the Real and Fake trials (see Figure 3).

### 3.2 Group comparisons for RPE based on extreme FI groups

**Figure 4:**
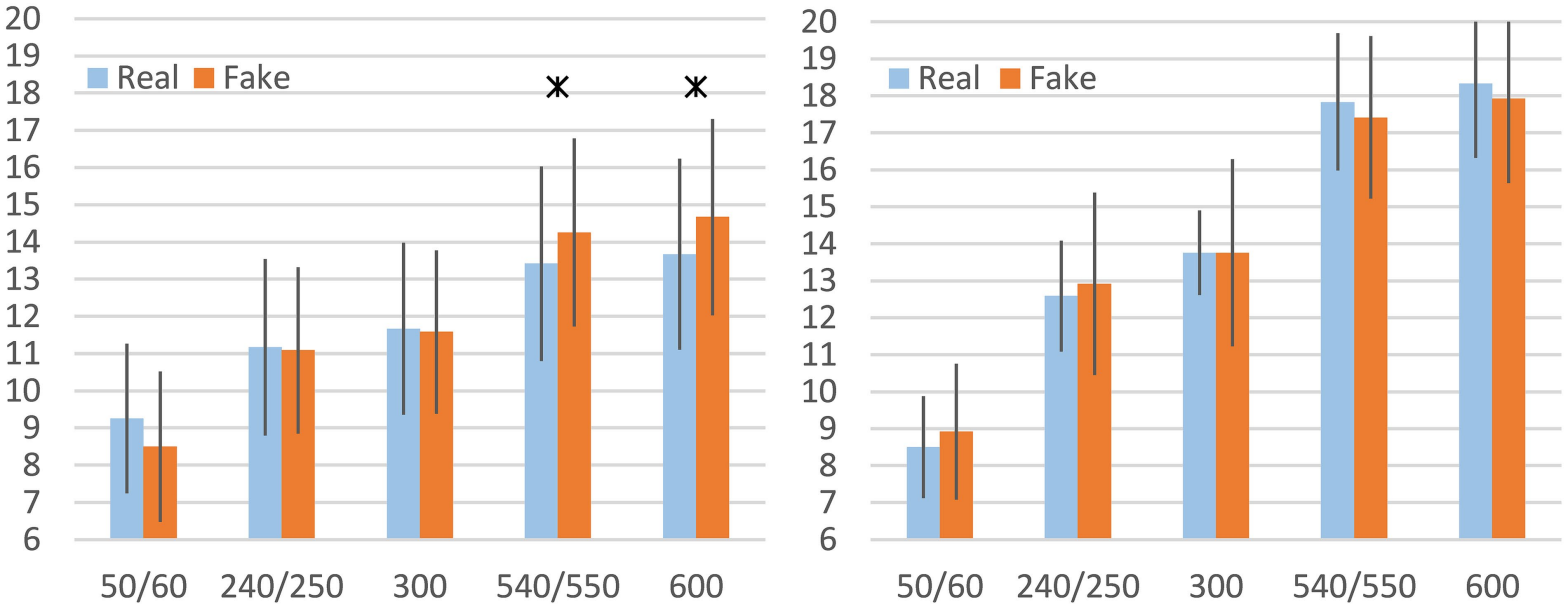
Mean rating of perceived exertion (RPE) for Real and Fake trials, based on the extreme FI groups, including standard deviation at comparable time points, left: good end, right: bad end; * indicates *p* < 0.05 and Cohen’s *d* > 0.5. Data are displayed as mean values ± SD.

For the RPE comparison, the classification of good end and bad end participants was based on FI. Significant differences were observed exclusively in the good end group (Figure 4). The mean RPE in the Fake trial was 0.9 points higher at 540/550 s and 1.0 point higher at 600 s compared to the Real trial.

## 4 Discussion

### 4.1 SEMG

In the analysis of physiologically measurable fatigue, statistically significant differences between the Real and Fake trials for MF L and MF R were only observed sporadically—specifically, on three occasions out of 40 tests within the “good end” group (MF L after the 1st and 2nd minute; MF R after the 4th minute). These differences appeared inconsistently, sometimes favoring the Real trial and other times the Fake trial, indicating a lack of systematic effect. All observed differences occurred before the 8th minute, suggesting that they were not related to the Real vs. Fake condition.

As trial order was both randomized and balanced for gender, time-related effects due to sequencing can be excluded. Hence, the observed variations are unlikely to be the result of systematic influences. Therefore, we conclude that the manipulated time announcement did not systematically affect FI changes in the back muscles, regardless of participants’ RPE.

One potential explanation lies in the physiological characteristics of back muscles, which are primarily postural. For instance, in the lumbar region, Type I muscle fibers account for approximately 60% of muscle cross-sectional area in men and about 70% in women (Mannion et al., 1997), making these muscles highly resistant to fatigue. Additionally, since the task did not involve dynamic, full-body exertion, cardiovascular strain played only a minor role. This is supported by the relatively low mean heart rate of 107 bpm at the end of the trial, in contrast to heart rates typically observed during ergometric or isokinetic exertion (100–150 bpm) (Scharf et al., 1994). Thus, muscular endurance was likely the primary limiting factor for fatigue. Furthermore, many participants did not reach complete exhaustion (i.e., Borg scale rating of 20 or voluntary termination).

Due to the unique design of the present study, comparable literature is limited. However, several studies have explored the effect of mental fatigue on SEMG parameters in other muscle groups. For example, Pageaux et al. induced mental fatigue via the AX-Continuous Performance Test (Cohen et al., 1996) and examined isometric endurance of the vastus lateralis and rectus femoris muscles using electrical stimulation (Pageaux et al., 2013). No difference in SEMG amplitude was found between groups. Similarly, Mehta and Parasuraman employed a subtraction task to induce mental fatigue and studied the flexor and extensor carpi radialis muscles (Mehta and Parasuraman, 2014), reporting no differences in maximum contraction time or SEMG parameters. Kowalski et al. used the Psychomotor Vigilance Test (Dinges and Powell, 1985) to induce mental fatigue and observed no SEMG amplitude differences in the tibialis anterior and medial gastrocnemius muscles (Kowalski et al., 2022).

Schouppe et al. investigated the impact of mental fatigue on trunk muscles, including the external and internal obliques, transversus abdominis, multifidus (MF), iliocostalis, and deltoid muscles of the dominant arm (Schouppe et al., 2019). However, rather than SEMG amplitude or frequency, they focused on anticipatory postural adjustment latency following rapid arm movements, and found no mental fatigue effect.

Although these studies differ methodologically, a consistent pattern emerges: SEMG parameters across various muscles appear unaffected by psychological interventions or stressors. These findings align with literature suggesting no significant link between mental fatigue and either peripheral or central muscle fatigue (Pageaux et al., 2015; Pageaux et al., 2013; Van Cutsem et al., 2017; Martin, 1981).

In addition, by comparing the FI changes between good and bad end groups it becomes evident, that different RPE levels are accompanied by the respective changes of physiological parameters, here exemplarily shown by the observed FI changes. These differences were not explicitly tested but are obvious. The same applies, if data were grouped according extreme RPE groups (see 4.2).

### 4.2 RPE

Across the entire sample, no significant differences in RPE were found between the Real and Fake trials. Therefore, the sample was subdivided based on FI into “good end” and “bad end” groups for further analysis.

**Figure 5:**
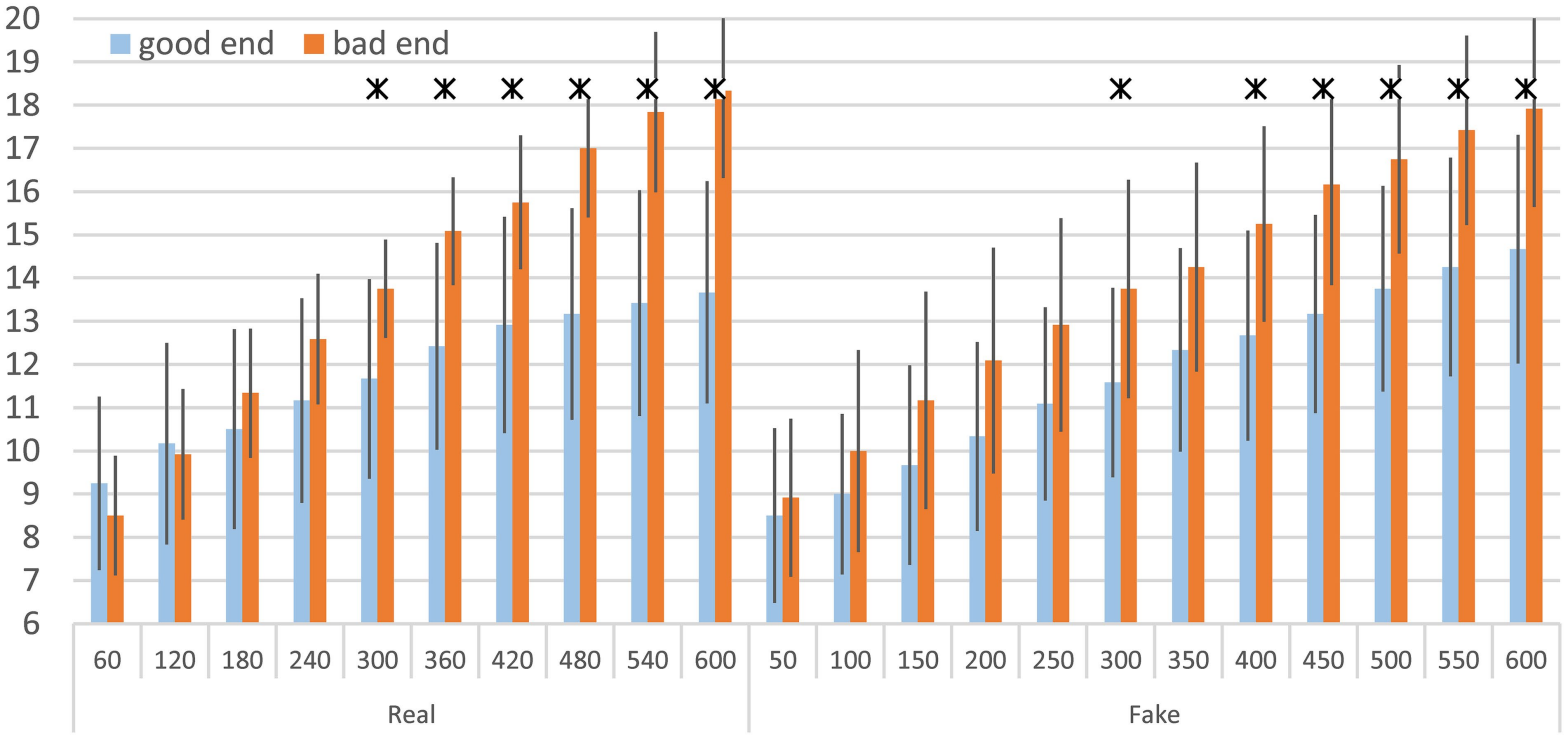
Mean rating of perceived exertion (RPE) for Real (left panel) and Fake trials (right panel), including standard deviation for good end and bad end; * indicates *p* < 0.05 and Cohen’s *d* > 0.5. Data are displayed as mean values ± SD.

Within-group comparisons revealed that only the good end group showed significantly higher RPE at the end of the Fake trial compared to the Real trial. When comparing RPE scores between the good and bad end groups at specific time points, the bad end group consistently reported higher exertion (see Figure 5), regardless of trial condition. Therefore, different amounts of physiologically determined fatigue are accompanied by corresponding RPE levels.

This further suggests that participants with lower levels of muscle fatigue were more susceptible to the manipulated trial conditions than those who were already highly fatigued. However, the subjective nature of the Borg scale likely contributed to a ceiling effect in the bad end group. These participants achieved an average of 18 by the end of the trial, potentially limiting the scale’s sensitivity to further changes. In contrast, the good end group averaged a score of 14 in the Real trial, leaving room for significantly higher scores in the Fake trial, thus enabling the detection of statistical differences.

Gender distribution may also have contributed to group differences. The good end group was 75% female, while the bad end group was 58.3% male. As women tend to have a higher proportion of Type I fibers in the back muscles (Mannion et al., 1997), they may experience less fatigue during isometric trunk muscle tasks, resulting in lower RPE scores. As we did not collect muscle samples, this remains hypothetical.

Previous studies on psychological factors and physical performance - whether involving whole-body endurance (Marcora et al., 2009; Pageaux, 2014) or localized muscular fatigue (Pageaux et al., 2013) - have shown that both physical and mental fatigue tend to elevate RPE (Pageaux and Lepers, 2016). However, those studies used different mental fatigue induction methods, typically involving 30–60 min cognitive tasks administered before the physical test, with participants being aware of the protocol. In contrast, our study involved real-time deception via RPE prompts, falsely suggesting a longer endurance task during execution. Whether this constitutes mental fatigue is debatable, but it clearly influenced RPE in less fatigued participants.

### 4.3 Limitations of the study

Several limitations must be acknowledged. High exertion levels caused excessive sweating, particularly in male participants, occasionally dislodging electrodes and introducing signal noise. Although these issues were addressed during testing (e.g., reattaching electrodes, drying sweat), they still required post-processing via artifact reduction routines.

Another limitation was the effectiveness of the deception in the Fake trial. After completing both trials, five participants indicated they suspected manipulation or attributed the timing change to technical issues. Some participants may have been so focused on the task that they failed to notice the discrepancy entirely. Further, participants were distracted from counting the time by indifferent small talk between the investigators.

Participant experience is also a confounding factor. Since the study excluded athletes and targeted the general German population, many participants were likely unfamiliar with such endurance testing. Some reported maximum exertion (RPE of 20) from minute 7 onward but continued for several more minutes, suggesting their internal exertion scales were underdeveloped, potentially biasing subjective RPE data.

Participants were not mentally stressed before the study, but the initial stress level was not explicitly detected. Therefore, the mental fatigue status remains unclear. As we only collected RPE values, we do not know, if participants may have developed different coping strategies with respect to the already mentioned self talking (Blanchfield et al., 2014). Verbal encouragement was also not standardized, but particularly provided if considered necessary, especially for the participants with high RPE ratings to prevent preterm task termination.

Finally, there may be a selection bias. Volunteers likely had higher-than-average motivation and willingness to engage in strenuous physical activity. The study announcement specifically mentioned trunk muscle endurance, which may have discouraged individuals with concerns about such tasks. Only two participants dropped out due to exhaustion, supporting this assumption. The recruitment primarily via the University Hospital Jena and social media channels also resulted in a younger, more homogenous sample (see Table 1), limiting generalizability but enhancing internal consistency.

### 4.4 Implications for research and practice

It remains unclear whether the more fatigued participants were genuinely unaffected by the manipulated RPE or whether their high exertion levels simply masked any effect due to a ceiling effect. Future studies should consider reducing task difficulty (e.g., 20° forward tilt) to avoid early exhaustion and better mimic everyday scenarios, where complete back muscle fatigue is rare. This would allow clearer differentiation in RPE responses and reduce ceiling effects. Ultimately, our results call for studies utilizing lower load levels to avoid ceiling effects due to limited subjective ratings for clearer differentiation of the impact of subjective (RPE) and objective (FI) fatigue related parameter changes. Additionally, other muscle regions should be investigated also, to find out if the actual results can be transferred to other body regions without restriction or have to be established per region.

As a side effect, the results also clearly showed that objective and subjective value levels describe the extent of fatigue similarly well. Therefore, ratings of perceived exertion during endurance tasks do reflect the amount of physiological fatigue related parameter changes, adequately, but are no prediction for task failure.

## 5 Conclusion

This study serves as a preliminary exploration of how psychological manipulations, specifically misleading time announcements, impact muscle fatigue and SEMG parameters during isometric endurance tasks involving the back muscles.

The results show that such manipulation had no consistent effect on fatigue-associated SEMG parameters across the full sample. Similarly, no significant differences in RPE were observed overall. However, when dividing participants based on fatigue levels, a clearer picture emerged: participants who were less fatigued showed increased RPE during the manipulated trial, while no effect was observed in the more fatigued group—possibly due to a ceiling effect.

These findings suggest that psychological interventions may influence perceived exertion, particularly in participants with moderate fatigue. They offer a foundation for future studies that should refine experimental design to better differentiate between physiological and psychological contributors to fatigue.

## Data Availability

The raw data supporting the conclusions of this article will be made available by the authors, without undue reservation.
